# Chemical Composition, and Antioxidant and Antimicrobial Activity of Oregano Essential Oil

**DOI:** 10.3390/molecules29020435

**Published:** 2024-01-16

**Authors:** Magdalena Walasek-Janusz, Agnieszka Grzegorczyk, Anna Malm, Renata Nurzyńska-Wierdak, Daniel Zalewski

**Affiliations:** 1 Department of Vegetable and Herb Crops, Faculty of Horticulture and Landscape Architecture, University of Life Sciences in Lublin, 51A Doświadczalna Street, 20-280 Lublin, Poland; magdalena.walasek@up.lublin.pl; 2 Chair and Department of Pharmaceutical Microbiology, Faculty of Pharmacy, Medical University of Lublin, 1 Chodzki Street, 20-093 Lublin, Poland; agnieszka.grzegorczyk@umlub.pl (A.G.); anna.malm@umlub.pl (A.M.); 3 Chair and Department of Biology and Genetics, Faculty of Pharmacy, Medical University of Lublin, 4a Chodzki Street, 20-093 Lublin, Poland; daniel.zalewski@umlub.pl

**Keywords:** *Origanum vulgare* L., GC/MS, carvacrol, antimicrobial activity, antioxidant activity

## Abstract

Antimicrobial resistance (AMR) is a global public health problem, and the rapid rise in AMR is attributed to the inappropriate and/or overuse of antibiotics. Therefore, alternative antimicrobial agents, including those of natural origin, are being sought for the development of new drugs. The purpose of our study was to analyze the chemical composition, and antimicrobial and antioxidant activities of four oregano essential oils (OEOs) from Poland, Europe, Turkey and the USA. The antimicrobial activity (AMA) was evaluated using 23 strains, including Gram-positive bacteria, Gram-negative bacteria and *Candida* species. The antioxidant activity (AA) of essential oils (EOs) was determined by the DPPH method. The main component of the EOs tested was carvacrol (76.64–85.70%). The highest amount of this compound was determined in the Polish OEO. The OEOs we tested showed antimicrobial resistance, which was especially strong against fungi (MIC = 0.06–0.25 mg/mL^−1^). These products also showed high AA (71.42–80.44%). OEOs high in carvacrol should be the subject of further research as potential antimicrobial and antioxidant agents.

## 1. Introduction

The presence of microorganisms in our environment, in food as well as on body surfaces and inside the body, can be both beneficial and harmful. These organisms can cause severe infections in humans and animals, and spoilage of food, and they can occur in places where their presence is undesirable (operating theatres, patient rooms, food processing plants, drug production, cosmetics, etc.) [1]. Effective control of pathogenic microorganisms has recently become increasingly difficult due, among other things, to the phenomenon of antibiotic resistance. Antimicrobial resistance (AMR) is a global public health problem, and the World Health Organisation has identified it as a significant health threat [1,2]. The rapid increase in AMR is attributed to the inappropriate and/or excessive use of antibiotics in human medicine, veterinary medicine and animal husbandry. For this reason, research into alternative agents has begun, using, among other things, raw materials of natural origin for these purposes. The development of new drugs now appears to be the most critical prerequisite for controlling microorganisms, which are increasingly resistant to previously used drugs [2]. Some of the more promising substances with potential antimicrobial activity are essential oils (EOs) obtained from various plant species [3,4]. The biological activity of EOs depends on various factors, including the extraction method. De J. Rostro-Alanis et al. [5], studying Mexican oregano (*Poliomintha longiflora* Gray), showed an increase in the content of some compounds following extraction by vacuum distillation, which increased antioxidant and antimicrobial activity.

*Origanum vulgare* L. (oregano, wild marjoram), a Mediterranean species of aromatic plant in the family Lamiacae, is a morphologically and chemically diverse species [6]. Oregano essential oil (OEO), which has been used in folk medicine since ancient times, exhibits antioxidant and antimicrobial activity [6,7]. The main component of OEO (more than 60%) is carvacrol, a monoterpenoid phenol present in smaller amounts in thyme or bergamot oil, among others. This compound has a wide variety of biological properties, including anti-inflammatory, antioxidant, anticancer, antimutagenic, analgesic, hepatoprotective, antiparasitic and antimicrobial activities. The broad spectrum of carvacrol activity, which extends to food spoilage or pathogenic fungi, yeasts and bacteria, as well as human, animal and plant pathogenic microorganisms, including drug-resistant and biofilm-forming microorganisms, is highlighted. The antimicrobial activity of carvacrol is attributed to its significant effect on the structural and functional properties of the cytoplasmic membrane [8,9,10,11]. Alkhafaji and Jayashankar [12] showed that oregano oil had optimal antimicrobial activity against all urinary pathogenic bacteria tested. A study by Aires et al. [13] demonstrated that thyme and oregano essential oils show promising antimicrobial activity, both in growth inhibition and cell destruction in the planktonic and biofilm states, which would be related to the presence of thymol and carvacrol.

OEOs may represent interesting therapeutic strategies for the treatment of multidrug-resistant pathogens. High variability in the chemical composition of OEOs extracted from different plant species, as well as commercial products, may affect their biological activity [10,11,12,13,14]. Our study aimed to characterise the chemical profile of four commercial OEOs from different regions (Turkey, USA, Poland, and Europe) and test their antimicrobial and antioxidant activities. In addition, we attempted to relate the chemical composition of OEOs to the biological activity of these oils by assessing the correlations between the percentage concentration of selected oil components and the MIC, MBC and MFC values obtained for these oils.

## 2. Results

### 2.1. Essential Oil Chemical Composition

A total of 119 compounds were determined in the OEOs studied, of which two were not identified (Table 1, Figures S1 and S2). The individual OEOs differed qualitatively and quantitatively. The highest number of compounds was determined in OEO1 (**96**) and the lowest in OEO3 (**54**). The dominant compound in all OEOs tested was carvacrol (76.64–85.70%), followed by o-cymene (3.61–5.00%), γ-terpinene (1.83–3.47%), β-bisabolene (0.02–3.00%), E-caryophyllene (0.98–2.93%), linalool (0.39–2.48%), thymol (1.65–2.43%) and borneol (0.26–1.35%). OEO3 contained the highest amount of carvacrol (85.70%), followed by o-cymene (5.00%), γ-terpinene (1.83%) and thymol (1.65%) in higher amounts. OEO1 and OEO4 contained similar amounts of carvacrol (77.73 and 77.61%, respectively), o-cymene (3.61 and 3.59%, respectively) and linalool (2.47 and 2.48%, respectively). OEO1 contained the most γ-terpinene (3.47%), borneol (1.35%), α-terpinene (1.09%) and myrcene (1.07%), while OEO4 stood out with the highest thymol content (2.43%).

Unlike the others, OEO1 did not contain sabinene or 1,8-cineole, and as for monoenes, it contained, among others, trans-sabinene hydrate, trans-piperitol, trans-carveol and neoiso-dihydro-carveol. There were also qualitative differences in other components. The manufacturer’s information on the chemical composition of the OEOs tested was mostly consistent with our results. The exceptions were OEO1, which contained less thymol (77.7%) than reported by the manufacturer (80%), and OEO2, which in turn, contained slightly more of this ingredient (76.6%) than reported by the manufacturer (60–75%).

**Figure 1:**
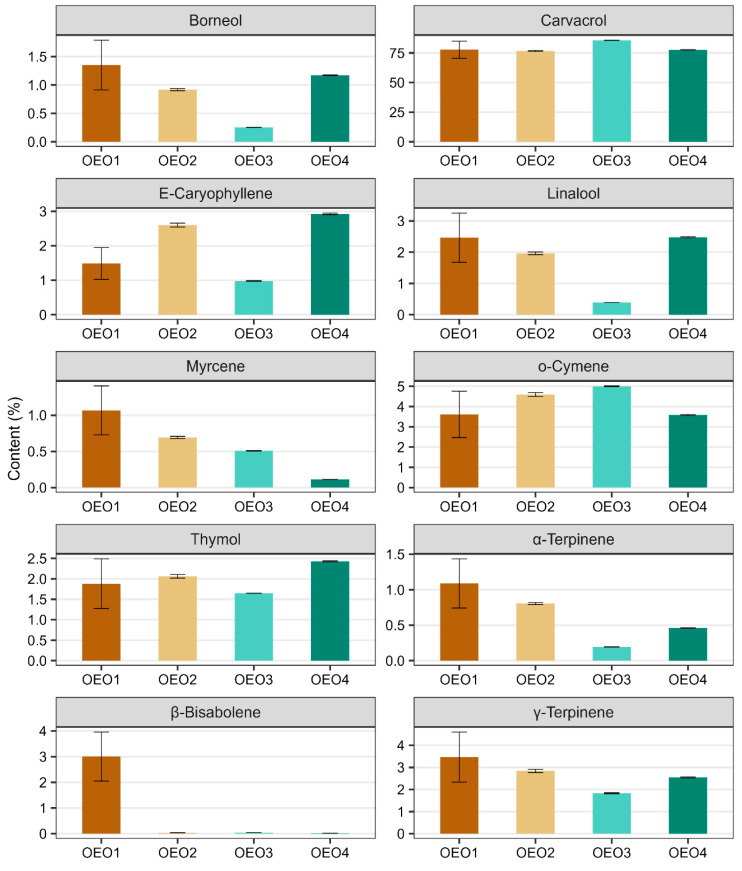
Content of the main compounds (compounds with a content greater than 1% in at least one OEO) identified in the studied oregano oils. Error bars mark standard deviations calculated from three technical replicates.

The data given in Figure 1 illustrate the contents of the ten main components of the OEOs (i.e., those whose content in at least one of the four oils is greater than 1%). In all the OEOs tested, carvacrol was the most abundant component, followed by o-cymene and γ-terpinene. It can be seen in the figure that β-bisabolene is only present in OEO1 (its presence in the other OEOs was well below trace amounts), while OEO3 contains the least γ-terpinene, borneol, caryophyllene, linalool, α-terpinene and thymol, but the most carvacrol and o-cymene. In addition, some compounds show a similar content pattern in the EOs tested, and this applies to compounds such as borneol and linalool, α- and γ-terpinene, and caryophyllene and thymol.

Based on the above summary, the chemical profile of all the OEOs tested can be described as high-carvacrol (>75%), including OEO1 as the borneol–linalool–myrcene–terpinene–bisabolol subtype, OEO2 as the E-caryophyllene–o-cymene subtype, OEO3 as the o-cymene subtype and OEO4 as the E-caryophyllene–linalool–thymol subtype. Considering the content of active compounds other than carvacrol, OEO1 stood out as having the richest chemical profile. In contrast, OEO3 was the least diverse in terms of composition, containing the most carvacrol (85.7%) and o-cymene (5.00%).

### 2.2. Antioxidant Activity (AA)

**Figure 2:**
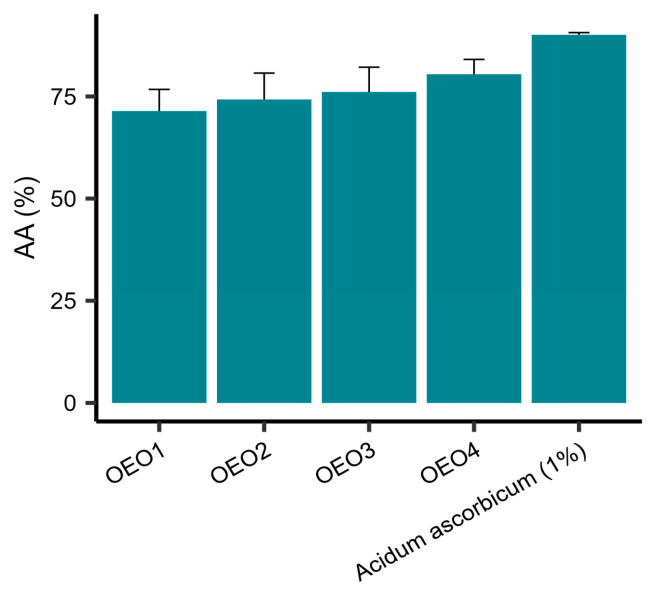
Antioxidant properties expressed as percentage reduction of DPPH radicals ± standard deviation for oregano essential oils. OEO—Oregano Essential Oil; OEO1 (Turkey), OEO2 (USA), OEO3 (Poland), OEO4 (Europe); AA—Antioxidant Activity.

The AA of the tested EOs was determined using DPPH as the free radical source. It was shown that all the tested EOs had a potent free radical scavenging capacity (Figure 2). The values of AA obtained were in the range 71.42–80.44%, with OEO4 showing the highest activity and OEO1 the lowest. The activities of OEO2 and OEO3 were similar. However, the activity of 1% vitamin C solution, as a control, was 90.03%.

### 2.3. Antimicrobial Activity

**Figure 3:**
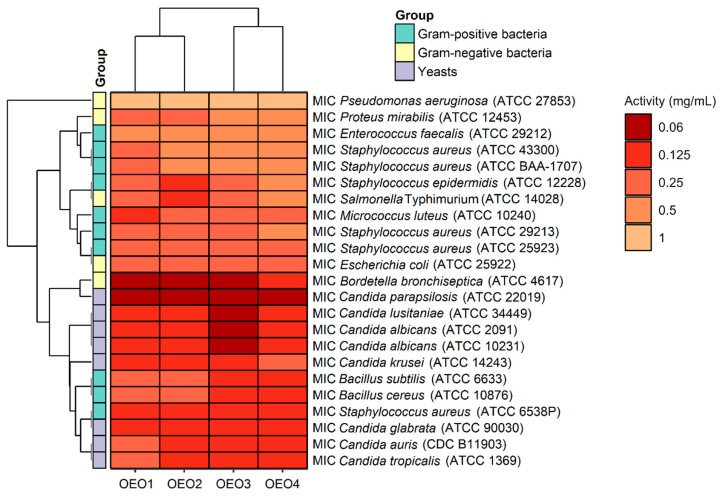
Antimicrobial activity (MIC doses) of oregano oils against tested microorganisms.

The antibacterial and antifungal activity results, including the MIC values, i.e., the lowest inhibitory concentration of the four tested OEOs (OEO1, OEO2, OEO3 and OEO4) obtained by the serial dilution method, are shown in Figure 3.

The OEOs showed varying activity against all bacteria, and the MICs were 0.06–1 mg/mL, with MIC values for Gram-positive and Gram-negative bacteria in the range 0.125–0.5 mg/mL and 0.06–1 mg/mL, respectively. In contrast, the MIC values for fungi were 0.06–0.25 mg/mL, suggesting that the fungal strains tested were more sensitive to OEOs than the bacterial strains. The highest activity among bacteria was found against *B. bronchiseptica* ATCC 4617 with MIC values of 0.06 mg/mL and 0.125 mg/mL for OEO1, OEO2, OEO3 and OEO4, respectively. All the EOs showed very good activity against *S. aureus* ATCC 6538P at 0.125 mg/mL. Excellent activity (MIC = 0.125 mg/mL) of OEO3, OEO4 and OEO2 was also shown against *B. cereus* ATCC 10876, *B. subtilis* ATCC 6633, *S. epidermidis* ATCC 12228, and *S.* Typhimurium ATCC 14028. All the EOs showed moderate activity (MIC = 0.5–1 mg/mL) against *P. aeruginosa* ATCC 27853 and *E. faecalis* ATCC 29212, while OEO3 and OEO4 showed activity against *P. mirabilis* ATCC 12453. In the case of two MRSA methicillin-resistant *S. aureus* strains (ATCC 43300 and ATCC BAA-1707), OEO2, OEO3, and OEO4 showed moderate activity (MIC = 0.5 mg/mL); good activity (0.25 mg/mL) was shown by the oil from Turkey (OEO1).

The MICs for the reference antimicrobial substances were as follows: the MIC of vancomycin for *S. aureus* ATCC 29213 was 1 μg/mL, the MIC of ciprofloxacin for *E. coli* ATCC 25922 was 0.5 μg/mL, and the MIC of fluconazole for *C. albicans* ATCC 10231 was 1 µg/mL. In contrast, the MICs of the OEOs were as follows: *S. aureus* ATCC 29213 (0.25–0.5 mg/mL), *E. coli* ATCC 25922 (0.25 mg/mL), and *C. albicans* ATCC 10231 (0.06–0.125 mg/mL). The reference substances showed better antimicrobial activity than the tested oils. It should be noted here that oils may contain compounds that cause an increase or inhibition of antimicrobial activity.

**Figure 4:**
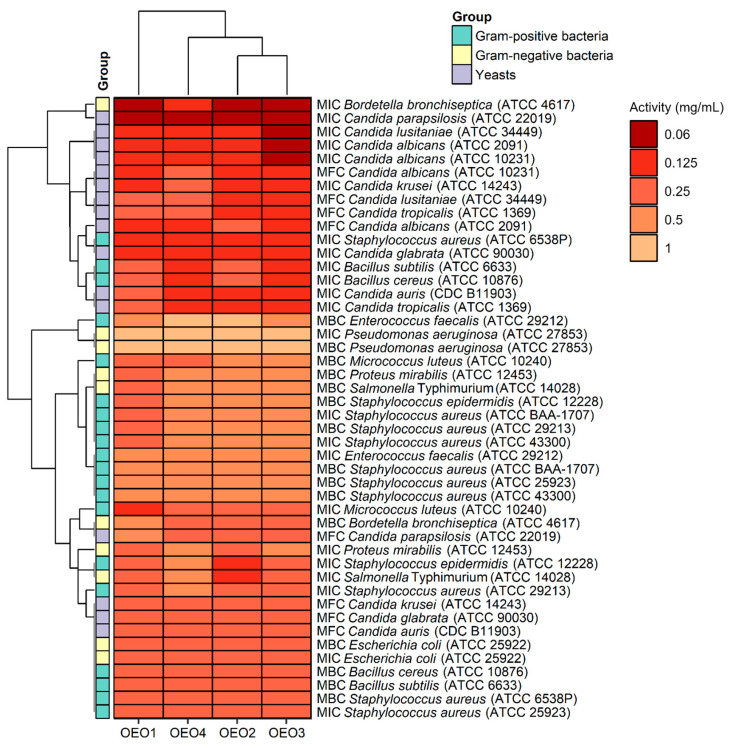
Antimicrobial activity (MIC, MBC, and MFC doses) of oregano oils against tested microorganisms.

The study also determined MBC and MFC values, which are in addition to the MIC values and indicate the potency of the lethal or static effect of the tested OEOs (Figure 4). The MBC or MFC is the lowest concentration of oils that kills 99.9% of bacteria or fungi over a fixed, slightly more prolonged period, such as 18 ± 2 h, under specified conditions. It was assumed that oils were bactericidal and fungicidal if the MBC or MFC values were less than four times the MIC (MBC/MIC or MFC/MIC ratio of ≤4) [15]. It was found that almost all the OEOs showed bactericidal and fungicidal activity due to MBC/MIC = 1–4 and MFC/MIC = 1–4, respectively, except for OEO1, which showed bacteriostatic activity (MBC/MIC = 8) against *B. bronchiseptica* ATCC 4617 and fungostatic activity (MFC/MIC = 8) against *C. parapsilosis* ATCC 22019.

Considering the data presented above, all the tested OEOs can be considered as promising sources of natural compounds with biocidal activity. Noteworthy is the strong antifungal activity (MIC = 0.06–0.125 mg/mL) of OEO3 against all tested Candida fungi capable of causing candidiasis, i.e., so-called opportunistic mycoses of the skin and mucous membranes and internal organs. It is also essential to highlight the strong antimicrobial activity (MIC = 0.06 mg/mL) of OEO1, OEO2 and OEO3 against *B. bronchiseptica*, which causes whooping cough-like infections, and the excellent antimicrobial activity (MIC = 0.125 mg/mL) of OEO3 and OEO4 against bacteria that cause food poisoning, such as *S. aureus* and *B. cereus*. OEO2, from the United States, showed very good activity (MIC = 0.125 mg/mL) against the opportunistic bacterium *S. epidermidis*, which can cause infections in immunocompromised patients, and against the absolutely pathogenic *S.* Typhimurium, which causes small and large intestinal inflammation and invasive food poisoning called salmonellosis.

**Figure 5:**
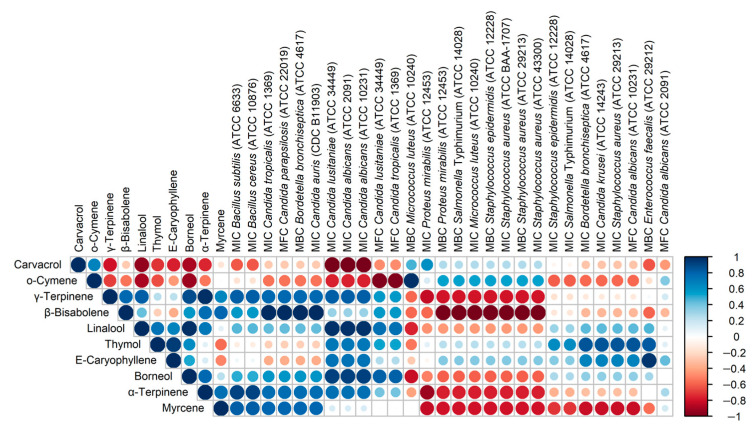
Corrplot presenting Pearson correlation coefficients for percentage content of 10 main constituents of studied essential oils and MIC/MBC/MFC doses determined for tested microorganisms. Microorganisms are ordered according to hierarchical clustering performed using maxima of Canberra distances.

Figure 5 presents the correlations between the content of the main components of the oils and the MIC/MBC/MFC values obtained for these oils. Bearing in mind that the analysis was performed on a small number of samples (4 OEOs), it is only possible to provisionally assess the directions of the correlation (positive/negative) and the strength of the correlation. The most interesting are negative correlations, i.e., those in which a higher content of an ingredient corresponds to lower MIC/MBC/MFC values, i.e., stronger antimicrobial activity. The presented data show that α-terpinene, γ-terpinene and β-bisabolene show a similar pattern of action, and myrcene also appears to be similar in this respect. Particular attention should be paid to the dominant carvacrol, the content of which is strongly negatively correlated with the MIC values for three strains of the Candida genus (*C. lusitaniae* ATCC 34449, *C. albicans* ATCC 2091 and *C. albicans* ATCC 10231). The data presented in Figure 5 also indicate strong correlations between some OEO components, probably resulting from common biosynthetic pathways, e.g., α-terpinene and γ-terpinene (R = 0.98), linalool and borneol (R = 0.98).

## 3. Discussion

### 3.1. Chemical Composition

OEOs are a mixture of numerous components, mainly mono- and sesquiterpenes. The proportions of individual compounds are genetically determined and form the basis for the classification of oregano species and chemotypes [16]. It should be added that OEO composition can be modified by ontogenetic factors [17], environmental factors [18], and production conditions [5,19]. Carvacrol, β-caryophyllene, o-cymene, γ-terpinene, linalool and thymol are mentioned as primary components of OEO [3,5]. Among the main constituents of OEO, carvacrol, a compound with broad biological activity, including antioxidant and antimicrobial activity, occupies a special place [9]. The commercial OEOs we studied were characterised by a high carvacrol content (>75%), as well as the presence of other highly active components (including o-cymene, E-caryophyllene, linalool, borneol, γ-terpinene, thymol, and myrcene). It should be noted that the characterisation of the OEOs reported by the manufacturer coincided with our results, which may be an example of good quality commercial products dedicated to health purposes.

### 3.2. Antioxidant Activity (AA)

Oregano is a good source of natural antioxidants with potential use in the food and pharmaceutical industries as a safer alternative to synthetic antioxidants [20,21]. The AA of the essential oil obtained from oregano herb is estimated by the DPPH method to be approximately 60–70% [3,14]. The stable DPPH radical is often used to determine the AA of single compounds as well as extracts obtained from plant material [22]. Using this method, we observed a high value of AA at the level of free radical reduction (>71%). A similarly high free radical scavenging capacity (77.2%) was obtained by Olmedo et al. [23] investigating an OE fraction containing carvacrol, terpinen-4-ol and γ-terpinene. The high AA of the essential oil obtained from *O. vulgare* L. ssp. *hirtum* was confirmed by three methods, including the DPPH method [22]. Interestingly, our study identifies the Europe-origin OEO4 as the strongest in terms of AA (77.61% of carvacrol), while the weakest (but still very strong) activity was shown by the Turkish-origin OEO1, with similar carvacrol content (77.73%). These results suggest the possible activity of other oil components, as well as synergistic actions. OEO1 was found to be the richest in active dominant compounds (borneol, linalool, myrcene, terpinene, and β-bisabolene), which may explain its strong AA.

OEO activity is associated with high content of carvacrol, thymol, or both [8,9,14]. This is due to the structure of the compounds, as carvacrol, like thymol, can trap free radicals and form stable forms that do not exhibit radical activity [8,9]. Both of these compounds, due to the presence of an aromatic ring, have a lower reducing potential compared with free radicals and are capable of donating hydrogen from the hydroxyl group attached to the aromatic ring, thus converting free radicals into inactive forms [20]. The OEOs studied contained more than 75% carvacrol, which presumably contributed to their high AA.

### 3.3. Antimicrobial Activity (AMA)

Essential oils show promise for antimicrobial activity, both in growth inhibition and cell destruction in the plankton and biofilm state [7,13]. Man et al. [10] found oregano, thyme, lemon and lavender oils to be the most active against microorganisms. Alexopoulos et al. [24] report that the mode of antimicrobial action of OEOs is disruption of the cytoplasmic membrane and cell wall. Regarding the antibacterial activity of the OEOs we tested, which were characterised by high carvacrol content (76.6–85.7%), we found the highest activity against *B. bronchiseptica* and *S. aureus*, as well as very good activity against *B. cereus*, *B. subtilis*, *S. epidermidis* and *S.* Typhimurium. We showed moderate activity of all OEOs against *P. aeruginosa*, *E. faecalis*, *P. mirabilis* and two MRSA strains of methicillin-resistant *S. aureus*. The high antimicrobial activity of OEO is emphasized by other authors. Studies by Man et al. [10] indicate that high-carvacrol OEO (80.5%) showed the best AMA and was the only oil tested that had bactericidal activity against all bacteria tested, including *P. aeruginosa* and methicillin-sensitive *S. aureus* (MRSA). Alkhafaji and Jayashankar [12] concluded that oregano oil has antimicrobial activity against *E. coli*, *P. aeruginosa*, *K. pneumoniae*, *P. mirabilis*, *Enterobacter aerogenes*, *Enterococcus faecalis*, *Acinetobacter baumannii*, *Neisseria gonorrhoeae*, *S. aureus*, and *S. epidermidis*, and may be suitable for medical applications, especially as an antibiotic against harmful microorganisms. Maggini et al. [25] report that carvacrol/thymol-rich OEO with a well-represented class of monoterpene hydrocarbons is a promising standardised antimicrobial herbal product. According to a study by Dolores Ibáñez et al. [7], oregano essential oil and its main constituent, carvacrol, were able to stop bacterial growth even at the lowest dose (1 μL). Carvacrol exerts antimicrobial activity against *S. aureus*, *P. aeruginosa*, coagulase-negative *Staphylococcus* spp., *Salmonella* spp., *Enterococcus* spp. *Shigella* spp. and *E. coli* [9]. Carvacrol and thymol showed antimicrobial activity against *S. enterica* and *S. aureus*. The synergistic effect of sodium chloride and essential oil containing carvacrol and thymol on *E. coli*, *Listeria monocytogenes* and *S. aureus* was demonstrated. The combined form of carvacrol/thymol (2.0 mM) with NaCl (≥3%) induced membrane rupture, with the result that the bacterial cells lost their ability to maintain osmotic equilibrium and were inactivated entirely [9]. This explains the broad AMA of the OEOs presenting a carvacrol chemotype which we tested.

The microbiological activity of oregano oils results from the fact that they have the ability to destabilize many vital functions of bacteria, including the biosynthesis of cell walls, proteins and nucleic acids. Authors from the Netherlands showed that the hydrophobicity of the compound may indicate that it is likely to disrupt the bacterial membrane, affecting the proton driving force and disrupting both the pH gradient and the flow of electrons across this membrane [26,27]. Additionally, research conducted by Nostro and Papalia [8] indicates that carvacrol significantly affects the structural and functional properties of the cytoplasmic membrane of bacteria. This is confirmed by our results regarding the antibacterial activity of the tested oregano essential oils.

The relationships between the carvacrol and thymol content of OEO and the AMA of OEO, therefore, appear to be well documented. It also seems to be of interest whether the other OEO components show similar properties and whether/how this affects the activity of the substance. The relationships we have reported between the contents of the main components of OEOs and their AMAs should be regarded as preliminary, requiring further research and confirmation on a more significant number of samples. It was important to note the excellent activity of OEO1 against two MRSA strains (ATCC 43300 and ATCC BAA-1707), which can be linked to the presence of carvacrol, terpinene and β-bisabolene. Carvacrol is responsible for the main antimicrobial activity and may act in synergy with other compounds to enhance this activity [5]. OEO1 was characterised by a high carvacrol content and the highest α- and γ-terpinene content (1.09 and 3.47%, respectively). This finding may support the thesis that carvacrol and γ-terpinene act synergistically and are mainly responsible for the antimicrobial activity of OEO [5]. Furthermore, OEO1 was distinguished by its significant content of β-bisabolene (3.0%), which was present in the other oils only in trace amounts (0.02–0.04%). Li et al. [28] report that (R)-β-bisabolene exhibited anti-adipogenic activity in the model organism *Caenorhabditis elegans* and antimicrobial activity selectively against Gram-positive bacteria. Our results and a preliminary evaluation of the direction of correlation between β-bisabolene content and the microbial activity of OEO against seven strains of Gram-positive bacteria (including MRSA) indicate that β-bisabolene may be as essential an antibacterial component as carvacrol.

The AMA of carvacrol extends to plant pathogens [3,9]. Essential oils of cinnamon, oregano and peppermint inhibited the growth of *F. oxysporum* at all doses tested (20, 10 and 5 μL) [7]. De Almeida et al. [3] showed that oregano oil (76% carvacrol) has a long-lasting fungistatic effect on *Aspergillus flavus* and *Penicillium commune*. The OEOs we analysed showed more potent antifungal activity than antimicrobial activity, confirming our previous results [4]. OEO3, which contained the most carvacrol and o-cymene, showed the highest activity (MIC = 0.06–0.125 mg/mL) against all *Candida* spp. fungi. The other oils also showed very good activity (MIC = 0.125–0.25 g/mL) against this group of microorganisms. The most sensitive fungus proved to be *C. parapsilosis* ATCC 22019, with a value of MIC = 0.06 mg/mL to all EOs. The high activity of OEO3 could be primarily attributable to its high carvacrol content (85.7%). This compound acts against *Candida* by altering the fungal cell membrane structure [29]. It is noteworthy that OEO3, distinguished by its highest concentration of carvacrol, showed potent activity against *B. bronchiseptica* ATCC 4617, *S. aureus* ATCC 6538P, *B. cereus* ATCC 10876, *B. subtilis* ATCC 6633, *S. epidermidis* ATCC 12228 and *S.* Typhimurium ATCC 14028, and against all *Candida* fungi. These results suggest that the high concentration of carvacrol had a significant effect on AMA OEO3 in this case. The antifungal effect of oregano oil is probably related to it is high carvacrol content. Data from the literature show that carvacrol has the ability to combine with sterols in the cytoplasmic membrane of fungi, causing its destabilization and thus limiting the basic life functions of these microorganisms [29]. Other authors showed that the antifungal mechanism of carvacrol resulted from its action on the cell membrane and destruction of the cytoplasm of hyphae, which ultimately led to the death of the mycelium [30].

## 4. Materials and Methods

### 4.1. Research Material

Four commercial oregano (*Origanum vulgare* L., Lamiaceae) essential oils (OEOs), designated OEO1 (from Turkey), OEO2 (from the USA), OEO3 (from Poland) and OEO4 (from Europe), were selected for this study. The details provided by the manufacturers for each OEO are provided in Table 2.

### 4.2. Essential Oil Isolation and Analyses

The samples were stored in the dark and at less than 4 °C. The chemical composition of the essential oils was determined by GC/MS. In the research, we used a GC apparatus (Shimadzu GC-2010 Plus, Merc, Kyoto, Japan) with a Zebron™ ZB-5MS column (length: 30 m; inner diameter: 0.25 mm; film thickness: 0.25 mm; Phenomenex Inc., Torrance, CA, USA). We used a temperature gradient of 50 °C for 3 min followed by an increase in 5 °C increments up to 250 °C. Helium with a flow rate of 0.5 mL min^−1^ was used as the carrier gas. Automatic dosing with a sample division (1 µL) with a division ratio of 1:100 was used in the analysis. The compounds were detected with a Vatran 4000 MS/MS detector. The mass spectrometer worked in the mass scanning range of 50–400 amu, while the scanning speed was 0.2 s scan^−1^. The mass spectrometer was operated in electron ionization (El) mode with an ionization energy of 70 eV. The ion source was maintained at 220 °C, while the interface and dispenser were maintained at 250 °C. Retention indices (Kovats) were calculated using a series of n-alkanes C6-C40. The qualitative analysis was performed based on MS spectra, comparing them with the spectra of the NIST library (62,000 spectra) and the LIBR terpene library (TR), provided by the Finnigan MAT company (Waltham, MA, USA). Data from the literature was used to confirm the identification of the compounds. In addition, the identification was also carried out based on comparisons of the obtained mass spectra with the NIST 2011 spectra library (National Institute of Standards and Technology, Gaithersburg, MD, USA), the MassFinder 2.1 computer spectra library (http://www.massfinder.com, accessed on 17 September 2022), and the mass spectra of reference compounds. Essential oil components are reported as a relative percentage of the total oil by peak area. Values ≥ 0.5 were considered significant.

### 4.3. Antioxidant Activity (AA) Determination by the DPPH Method

The antioxidant activity (AA) assessment was carried out for four essential oils using the DPPH method according to the method given by Yen and Chen [31]. This method consists of the colourimetric measurement of the degree of reduction of DPPH free radicals (2,2-diphenyl-1-picrylhydrazyl, Merck, Poznań, Poland). Absorbance was measured using a Hitachi U-2900 UV–Vis model spectrophotometer. The absorbance of the solutions was measured at a wavelength of 517 nm using the reference solution (methanol). In this study, we used a 1% methanol solution of vitamin C as a control. To determine DPPH activity, 10 μL was added to a sample with methanol and DPPH reagent and used for testing. The analysis was performed three times. To determine DPPH activity, 50 μL was added to a sample with methanol and DPPH reagent and used for testing. Decreasing absorbance levels indicated increased antioxidant activity of the essential oils. The experiments were performed in triplicate and the results are expressed as the percentage of DPPH inhibition according to the formula given by Rossi et al. [32]:

X% = 100 − [At − (Ar × 100)],

where X% is the percent reduction of DPPH radicals, At is the absorbance of the test sample, and Ar is the absorbance of the blank.

### 4.4. Antimicrobial Activity (AMA)

The double-dilution method recommended by EUCAST (European Committee on Antimicrobial Susceptibility Testing) [33], modified by Malm et al. [34], was used to test the AMA of the four oregano essential oils (OEO1, OEO2, OEO3 and OEO4). Strains from the American Type Culture Collection (ATCC) were used as indicator microorganisms. These included Gram-positive bacteria (*Staphylococcus aureus* ATCC 25923, *S. aureus* ATCC 6538P, *S. aureus* ATCC 29213, *S. aureus* ATCC BAA-1707, *S. aureus* ATCC 43300, *S. epidermidis* ATCC 12228, *Micrococcus luteus* ATCC 10240, *Bacillus subtilis* ATCC 6633, *B. cereus* ATCC 10876, and *Enterococcus faecalis* ATCC 29212), Gram-negative bacteria (*Escherichia coli* ATCC 25922, *Salmonella* Typhimurium ATCC 14028, *Bordetella bronchiseptica* ATCC 4617, *Proteus mirabilis* ATCC 12453, and *Pseudomonas aeruginosa* ATCC 27852), and fungi (*Candida albicans* ATCC 10231, *C. albicans* ATCC 2091, *C. auris* CDC B11903, *C. glabrata* ATCC 90030, *C. krusei* ATCC 14243, *C. parapsilosis* ATCC 22019, *C. lusitaniae* ATCC 34449, and *C. tropicalis* ATCC 1369). All the bacteria were cultured on Mueller–Hinton Agar (MHA), while the fungi were cultured on RPMI 1640 Agar (RPMI 1640A) and incubated at 35 ± 1 °C for 18 ± 2 h. Subsequently, the inoculum was prepared from the grown colonies in sterile saline at a density of 0.5 McFarland, corresponding to 1.5 × 108 CFU (colony forming units)/mL for bacteria and 5 × 106 CFU/mL for fungi. All the oregano essential oils (OEO1, OEO2, OEO3 and OEO4) were dissolved in DMSO to a final concentration of 100 mg/mL. Then, in 96-well titer plates, they were double-diluted in Mueller–Hinton liquid medium (MHB) for bacteria, or in RPMI 1640 liquid medium (RPMI 1640B) for fungi, to final concentrations of 1 to 0.06 mg/mL.

Then, 2 µL of a specific bacterial or fungal inoculum was added to each well containing 200 µL of double dilutions of the oils in the appropriate culture medium. After incubation at 35 ± 1 °C for 18 ± 2 h, the MIC (minimum inhibitory concentration) was assessed, using a BioTek (Winooski, VT, USA) spectrophotometer, as the lowest concentration of oils showing complete inhibition of bacterial or fungal growth. The MBC (minimum bactericidal concentration) or MFC (minimum fungicidal concentration) was determined by taking 5 µL of bacterial or fungal culture from each well and plating it on MHA or RPMI 1640 Agar for bacteria and fungi, respectively. The plates were incubated at 35 ± 1 °C for 18 ± 2 h. The lowest concentrations of oils without visible bacterial or fungal growth were assessed as MBC or MFC, respectively. Vancomycin (range 0.06–16 μg/mL) and ciprofloxacin (range 0.015–16 μg/mL) were used as reference antimicrobial substances to determine the activity against Gram-positive and Gram-negative bacteria, while fluconazole (range 0.06–16 μg/mL) was used as a reference for antifungal activity.

The following control systems were used in the study: The first was a sterility control of MHA and MHB media and RPMI 1640A and RPMI 1640B containing neither the test oils nor the reference microorganisms. The second was a control of all oils at concentrations from 1 to 0.06 mg/mL on MHB and RPMI 1640B media, and the third was a fertility control of MHA and MHB media, and RPMI 1640A and RPMI 1640B media with all the test reference microorganisms but no oils. The experiments were carried out in triplicate. Of the three MIC values, MBC and MFC are shown as the most typical representative values.

### 4.5. Data Analysis and Visualisations

Data analysis was carried out using the R 4.3.1 programming environment (https://www.r-project.org, accessed on 28 November 2023) and appropriate packages. The bar plot that presents the percentage content of the main essential oil components was created using the ggplot2 3.4.3 package (https://cran.r-project.org/web/packages/ggplot2/index.html, accessed on 10 January 2024). The pheatmap 1.0.12 (https://cran.r-project.org/web/packages/pheatmap/index.html, accessed on 28 November 2023) and the ggplot2 3.4.3 packages were used to draw a heatmaps illustrating the antimicrobial activity of the analysed essential oils. Data on the heatmaps were ordered using the hierarchical clustering of the average values of Canberra distances. The relationships between the percentage content of the main compounds and the antimicrobial activity of oregano essential oils were evaluated using a correlation analysis, which was performed using the Pearson algorithm implemented in the base cor function in R. If the same values of antimicrobial activity were obtained for all OEOs, these data were excluded from the correlation analysis. The results of the correlation analysis are presented as a correlation plot drawn using the corrplot 0.92 package (https://cran.r-project.org/web/packages/corrplot/index.html, accessed on 28 November 2023).

## 5. Conclusions

OEOs are highly diverse and biologically active EOs whose AMA and AA depend on their chemical profile. The AA and AMA of OEOs are probably related to the different proportions of volatile substances that may be responsible for limiting bacterial growth and inactivating free radicals. These actions may be due to the activity of individual compounds, synergistic actions, or both. The OEOs we analysed represented a high-carvacrol chemotype and varied in terms of the other dominant components.

The OEOs showed strong AA as well as pronounced activity against fungi and bacteria. The *Candida* strains tested were more sensitive to the OEOs than the bacterial strains. The most sensitive strain to all OEOs was *C. parapsilosis* ATCC 22019. The OEO containing the most carvacrol and o-cymene showed the highest activity against all *Candida* fungi. The most potent activity among the bacteria was found against *B. bronchiseptica* ATCC 4617. For two MRSA strains (ATCC 43300 and ATCC BAA-1707), one of the OEOs tested showed good activity, and three showed moderate activity. Preliminary evaluation of the direction of correlation between the components and the microbial activity of the oil yielded promising results, which need to be confirmed in further studies.

Our results indicate the high biological potential of OEO and the need for further research to identify fractions and/or components of OEO targeting antimicrobial and antioxidant activities. Particular attention would need to be paid to β-bisabolene, as well as possible synergistic actions of other active OEO components.

## Figures and Tables

**Table 1:** The chemical composition of the OEOs.

Compound	RI	RT (min)	Content (%)			
			OEO1	OEO2	OEO3	OEO4
Tricyclene	921	6.159	0.01	0.01	-	0.01
α-Thujene	924	6.247	0.20	0.56	0.05	0.08
α-Pinene	932	6.467	0.49	0.52	0.88	0.16
α-Fenchene	947	6.91	-		0.04	-
Camphene	948	6.957	0.21	0.13	0.27	0.15
Thuja-2,4(10)-diene	952	7.073	0.01	-	-	-
5-methyl-Furfural	961	7.326	0.01	-	-	-
Sabinene	971	7.649	-	0.01	0.07	0.01
β-Pinene	977	7.809	0.07	0.67	0.25	0.47
1-Octen-3-ol	979	7.877	0.08	0.14	0.04	0.17
trans-meta-Mentha-2,8-diene	983	7.983	-	0.02	0.02	-
3-Octanone	985	8.049	-	0.06		0.08
Myrcene	989	8.166	1.07	0.69	0.51	0.11
3-Octanol	997	8.421	-	0.04		0.05
δ-2-Carene	1004	8.642	-	0.04	0.03	-
α-Phellandrene	1006	8.724	0.23	0.07	0.01	0.04
p-Mentha-1(&),8-diene	1008	8.802	0.08	0.01	0.01	0.01
α-Terpinene	1015	9.076	1.09	0.81	0.19	0.46
p-Cymene	1017	9.168	0.01	0.01	0.03	0.01
o-Cymene	1022	9.346	3.61	4.59	5.00	3.59
Limonene	1026	9.493	0.19	0.55	0.21	0.58
β-Phellandrene	1027	9.546	0.25	-	0.07	-
1.8-Cineole	1029	9.599	-	0.74	0.32	0.85
(Z)-β-Ocimene	1032	9.719	0.04	0.01	0.03	0.01
M.IN.-β-Ocimene	1041	10.077	0.08	0.02	0.01	0.01
γ-Terpinene	1052	10.500	3.47	2.84	1.83	2.55
cis-Sabinene hydrate	1063	10.915	0.14	0.02	0.02	0.02
Terpinolene	1076	11.437	0.22	0.05	0.09	0.02
p-Cymenene	1081	11.619	0.05	0.01	0.01	0.01
Linalool	1090	11.954	2.47	1.97	0.39	2.48
trans-Sabinene hydrate	1093	12.062	0.01	-	-	-
endo-Fenchol	1109	12.64	-	-	-	0.01
cis-p-Menth-2-en-1-ol	1115	12.825	0.04	0.01	-	0.01
α-Campholenal	1118	12.916	0.05	-	-	-
γ-Terpineol	1119	12.942	-	-	0.01	-
trans-Sabinol	1134	13.405	0.05	0.01	0.01	-
neo-3-Thujanol	1136	13.477	0.06	-	-	0.01
Camphor	1142	13.644	-	0.61	-	0.76
cis-Chrysanthenol	1144	13.735	0.01	-	-	-
iso-Menthone	1151	13.947	-	0.02	-	-
β-Pineneoxide	1164	14.349	-	-	0.01	-
Borneol	1170	14.532	1.35	0.92	0.26	1.17
Terpinen-4-ol	1179	14.825	0.97	0.44	0.16	0.55
p-Cymen-8-ol	1187	15.073	0.03	-	-	0.01
α-Terpineol	1196	15.349	0.11	0.26	0.04	0.33
γ-Terpineol	1198	15.410	0.21	0.47	0.09	0.54
trans-dihydro Carvone	1208	15.737	-	0.01	-	0.01
trans-Piperitol	1214	15.938	0.02	-	-	-
trans-Carveol	1224	16.273	0.03	-	-	-
neoiso-dihydro Carveol	1229	16.452	0.05	-	-	-
Thymol, methylether	1241	16.840	0.15	0.27	0.16	0.33
Cumin aldehyde	1246	17.024	-	0.05	-	0.04
Carvone	1251	17.224	0.33	0.01	0.01	0.02
cis-Myrtanol	1257	17.402	-	0.01	-	-
Carvotan acetone	1258	17.434	0.06	-	0.01	0.01
2E-Decenal	1268	17.781	-	0.01	-	0.01
Carvenone	1271	17.854	0.07	-	-	-
2E-Decen-1-ol	1276	18.041	-	-	-	0.01
Thymol	1295	18.689	1.88	2.06	1.65	2.43
Carvacrol	1309	19.152	77.73	76.64	85.70	77.61
NI	1319	19.505	0.23	-	-	-
methyl-Anthranile	1344	20.345	-	-	-	0.01
δ-Elemene	1349	20.498	-	0.05	-	0.06
NI	1355	20.701	-	0.09	-	0.12
Nerylacetate	1360	20.888	0.04	-	-	-
Carvacrol acetate	1367	21.097	0.36	0.01	-	-
α-Ylangen	1370	21.226	0.01	-	-	-
Isoledene	1372	21.262	0.01	-	-	-
α-Copaene	1377	21.433	0.07	0.07	-	0.08
Geranylacetate	1380	21.533	0.07	0.01	-	0.01
β-Bourbonene	1384	21.696	0.06	0.01	-	-
β-Elemene	1390	21.88	-	0.01	-	0.01
Z-Jasmonene	1395	22.044	0.01	-	-	-
Methyl eugenol	1402	22.292	0.09	0.01	-	-
α-Funebrene	1405	22.364	-	-	-	0.01
α-Gurjunene	1408	22.455	0.01	-	-	-
E-Caryophyllene	1423	22,856	1.49	2.60	0.98	2.93
β-Copaene	1432	23,100	0.02	0.01	0.01	0.01
β-Gurjunene	1435	23,181	0.01	0.01	0.01	0.01
trans-α-Bergamotene	1438	23.275	0.09	-	-	-
α-Guaiene	1440	23.335	-	-	-	0.01
Aromadendrene	1444	23.451	0.35	-	0.01	-
α-Himachalene	1457	23.788	0.03	0.01	-	0.01
α-Humulene	1464	23.988	0.09	0.53	0.01	0.58
allo-Aromadendrene	1469	24.122	0.05	0.01	-	0.01
trans-Cadina-1(6),4-diene	1482	25.502	0.03	0.01	-	-
α-Cuprenene	1486	24.596	-	-	-	0.01
allo-Aromadendrene	1469	24.129	-	-	0.01	-
γ-Muurolene	1486	24.600	0.06	0.00	0.01	-
α-Amorphene	1491	24.735	0.02	-	-	-
γ-Amorphene	1496	24.877	0.02	-	-	-
Viridiflorene	1503	25.109	0.21	-	-	-
α-Muurolene	1508	25.268	0.05	0.01	-	-
Epizonarene	1510	25.342	0.04	-	-	-
trans-β-Guaiene	1515	25.480	0.03	-	-	-
β-Bisabolene	1519	25.616	3.00	0.04	0.04	0.02
γ-Cadinene	1524	25.782	0.23	0.01	0.01	-
δ-Cadinene	1528	25.938	0.20	0.01	0.02	0.01
trans-Calamenene	1531	26.039	-	-	-	0.01
β-Sesquiphellandrene	1533	26.089	0.07	-	-	-
trans-Cadina-1,4-diene	1541	26.361	0.02	-	-	-
α-Cadinene	1545	26.493	0.03	-	-	-
Germacrene B	1568	27.254	0.01	-	-	-
Spathulenol	1581	27.695	0.25	-	0.01	-
Caryophyllene oxide	1585	27.850	0.46	0.14	0.45	0.37
Globulol	1588	27.954	0.03	-	-	-
β-Atlantol	1610	28.582	0.01	-	-	-
Humulene epoxide II	1613	28.661	0.02	0.02	-	0.05
1,10-di-epi-Cubenol	1620	28.824	0.05	-	-	-
Eremoligenol	1640	29.326	0.03	-	-	-
Caryophylla-4(14),8(15)-dien-5-alpha-ol	1646	29.451	0.02	-	0.01	0.01
α-Muurol	1651	29.584	0.28	-	-	-
14-hydroxy-9-epi-M.IN.-Caryophyllene	1656	29.712	0.05	-	-	-
Eremoligenol	1667	29.976	0.09	-	0.01	0.01
(Z)-α-Santalol	1684	30.402	0.03	-	-	0.01
Khusinol	1689	30.516	0.01	-	-	-
α-Bisabolol	1701	30.814	0.02	-	-	-
(Z)-α-trans-Bergamotol	1703	30.860	0.02	-	-	-
8-Cedrene-13-ol	1706	30.967	0.02	-	-	-

OEO—Oregano Essential Oil; OEO1 (Turkey), OEO2 (USA), OEO3 (Poland), OEO4 (Europe); RI—Retention Index; RT—Retention Time; NI—Compound Not Identified with a Specific Mass Spectrum.

**Table 2:** Characteristics of the oregano essential oils (OEOs) tested (manufacturers’ data).

Essential Oil	Origins Manufacturer	Characteristics of the Oil
OEO1	Turkey (Hepatica)	The oil is derived from the wild oregano (*O. vulgare* L.) plant, a carvacrol chemotype. It has a high concentration of carvacrol (80%) and a low concentration of thymol (<2%). A daily portion (2 drops) contains 60 mg of the oil. Pure oil, 100%.
OEO2	USA (Young Living)	The oil has an intense, pungent aroma; strong immune system-boosting properties, and supports the respiratory system. The oil contains 60–75% carvacrol.
OEO3	Poland (Medi-Flowery)	Oil obtained from organically grown oregano (*O. vulgare* L.) plants, 100% oregano oil. Carvacrol content minimum 80%. The product is certified for organic farming.
OEO4	Europe * (Bamer)	Top quality, pure oil extracted from oregano leaves. Laboratory-tested. Manufacturer does not provide information on chemical composition.

* No country-of-origin information.

## Data Availability

Data are contained within the article and supplementary materials.

## Supplementary Materials

The following supporting information can be downloaded at: https://www.mdpi.com/article/10.3390/molecules29020435/s1. Figure S1: Chromatogram of oregano essential oils (OEO1 and OEO2); Figure S2: Chromatogram of oregano essential oils (OEO3 and OEO4).
