# Supplementary figures and images for: Chemical Composition, and Antioxidant and Antimicrobial Activity of Oregano Essential Oil
Source: Molecules. 2024 Jan 16;29(2):435. doi: 10.3390/molecules29020435 (PMC10818459; doi:10.3390/molecules29020435)

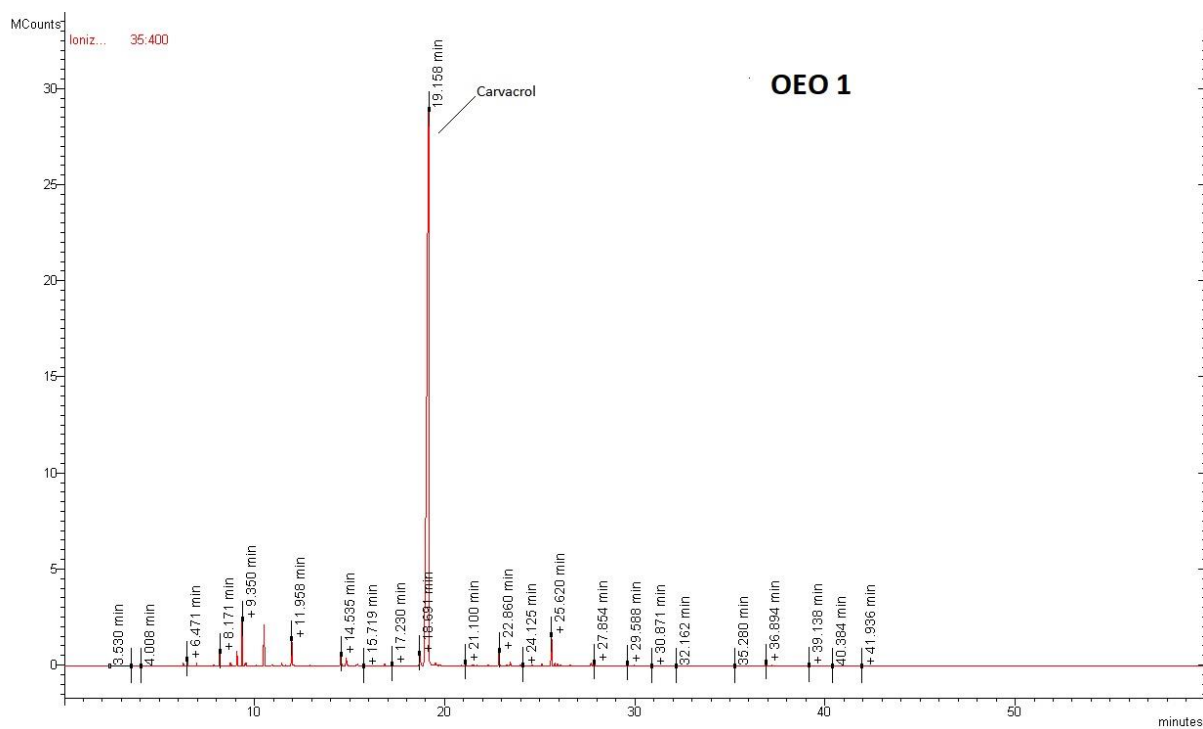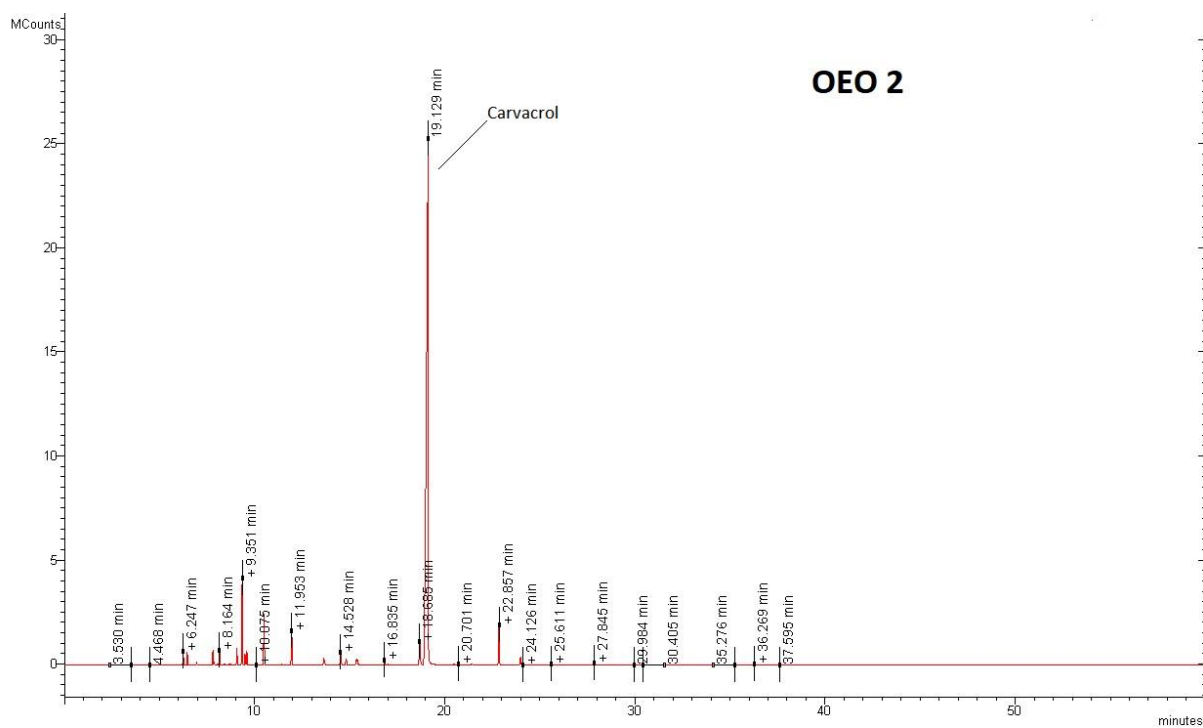

Figure S1. Chromatogram of oregano essential oils (OEO1 and OEO2)

Supplement: Supplementary file 1 [file molecules-29-00435-s001.zip › Figure S1.pdf]

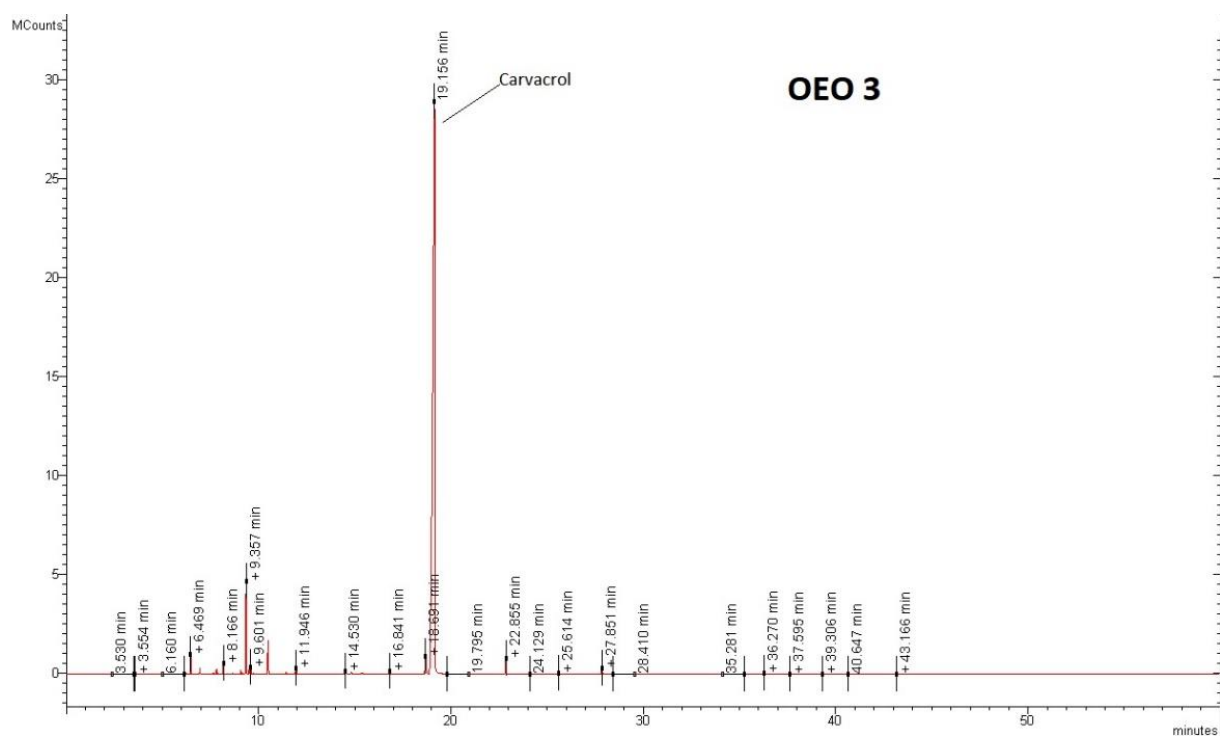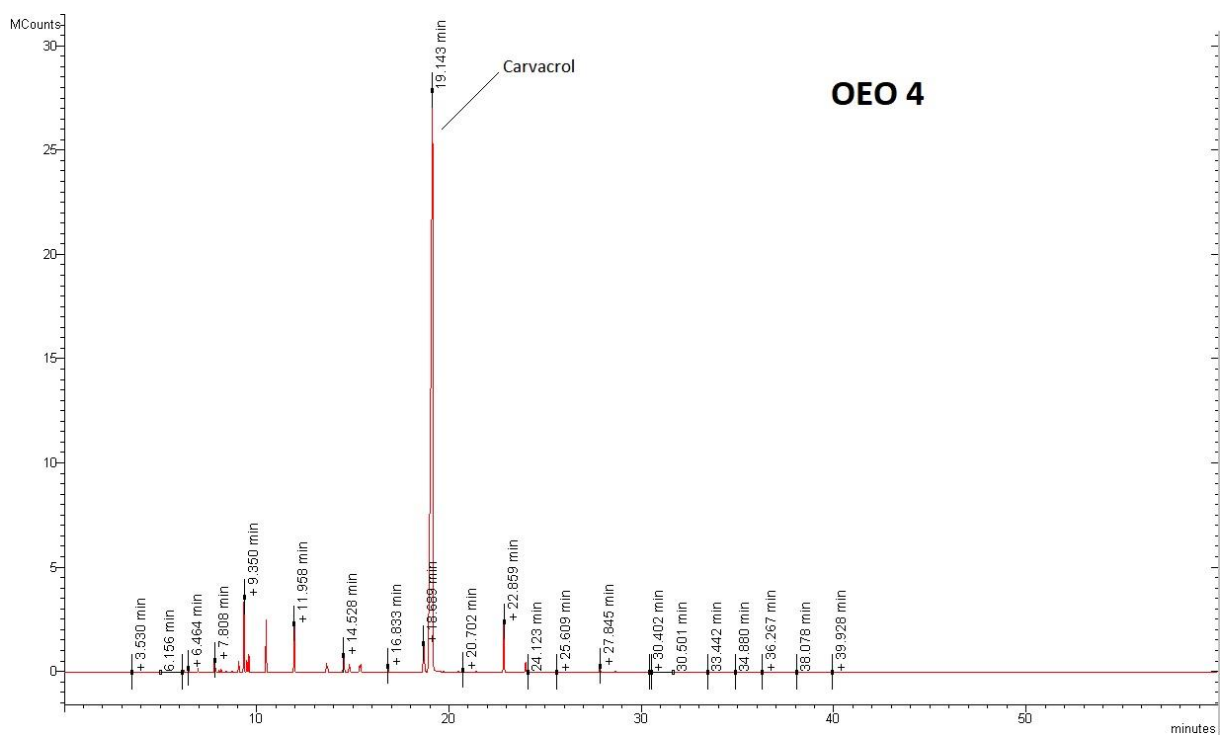

Figure S2. Chromatogram of oregano essential oils (OEO3 and OEO4)

Supplement: Supplementary file 1 [file molecules-29-00435-s001.zip › Figure S2.pdf]
